# A rare initial presentation of a thymic neuroendocrine tumor as Cushing's syndrome

**DOI:** 10.1002/ccr3.4435

**Published:** 2021-07-09

**Authors:** Ahmad S. Matarneh, Abdelrahman O. Hamad, Mohammad K. Hamad, Elhadi B. Elouzi, Nabil S. Mahmood, Khaled Al‐Sawalmeh, Issam Al‐Bozom, Mousa S. Hussein, Mohamed A. Yassin

**Affiliations:** ^1^ Department of Internal Medicine Hamad Medical Corporation Doha Qatar; ^2^ Department of Endocrinology Hamad Medical Corporation Doha Qatar; ^3^ Department of Radiology Hamad Medical Corporation Doha Qatar; ^4^ Department of Laboratory Medicine and Pathology Hamad Medical Corporation Doha Qatar; ^5^ Department of Pulmonology Hamad Medical Corporation Doha Qatar; ^6^ Department of Medical Oncology Hamad Medical Corporation Doha Qatar

**Keywords:** Cushing's syndrome, hypercortisolism, Neuroendocrine neoplasm, pituitary incidentaloma, thymic malignancies

## Abstract

While evaluating the cause of Cushing's syndrome, biochemical confirmation should be sought first as imaging studies might misdirect the diagnosis toward the wrong problem. One of the rare secondary causes that should be kept in mind while evaluating Cushing's syndrome is the thymic neuroendocrine tumor.

## Graphical abstract

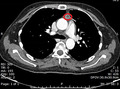

## 1 INTRODUCTION

Thymic neuroendocrine tumors represent a rare group of neoplasms, with only a few cases described in the literature. They are considered malignant which have the propensity to metastasize. The presentation can be variable as it can present with local symptoms, or if functional, it can secrete hormones, leading to paraneoplastic syndromes such as acromegaly and Cushing's syndrome. Cushing's syndrome is characterized by the signs and symptoms of exposure to excess glucocorticoids. Establishing the diagnosis is often challenging as the signs and symptoms may not always be clear. Among several benign and malignant causes, ectopic secretion of ACTH by nonpituitary tumors accounts for about 10‐15 percent of Cushing's syndrome. Most ectopic cases are caused by neuroendocrine tumors arising from the lung, pancreas, or thymus. Due to the diagnostic and presentation challenges, it can often be missed. We wrote this report to highlight a diagnostic dilemma we encountered and the association of thymic neuroendocrine tumors with Cushing's syndrome, along with the patient's outcome. We report a 24‐year‐old Filipina woman who was presented to our hospital with generalized fatigue and weakness. She was found to have an insuppressible low dose of dexamethasone test, which was consistent with Cushing's syndrome, and further evaluation revealed a pituitary microadenoma; however, the biochemical profile was compatible with an ectopic source and found to have a thymic neuroendocrine tumor, which was treated with surgical excision by video‐assisted surgery. The patient had an uncomplicated recovery course with normalization of her blood pressure and hormonal profile. Evaluation for Cushing's syndrome should always be started with biochemical confirmation and then imaging to look for a source as seeking imaging first can be misleading. Thymic neuroendocrine malignancies are a rare group of neoplasms that can be present with Cushing's syndrome, and they should be kept in mind while dealing with an ectopic Cushing's syndrome. The mainstay of treatment is surgery, with good overall survival and outcomes.

Cushing's syndrome is a syndrome that manifests with signs and symptoms of hypercortisolism and usually affects multiple systems. Depending on the level and duration of exposure to the high cortisol levels, the presentation varies widely, with the most observed findings being obesity, round (moon) faces, acne, high blood pressure, and poorly controlled blood sugar with multiple electrolyte derangements.^1^ Moreover, the presentation might differ between men and women as men can present at a younger age than females, who usually present at a later age, with symptoms of gonadal dysfunction such as amenorrhea and other menstrual abnormalities being more common.^2^

Diagnosis usually requires a high clinical suspicion. It is generally divided into two steps. The first step is to establish the presence of a hyper‐cortisol state with confirmatory laboratory studies, such as high cortisol levels and an insuppressible cortisol level with the administration of low‐dose dexamethasone (1 mg). The second step is to identify the source, which is carried out by administering high‐dose dexamethasone (8 mg); failure to suppress cortisol points toward an ectopic source of ACTH secretion.^3^

The treatment choice depends on the underlying etiology. One of the secondary causes of Cushing's syndrome is neuroendocrine tumors of the thymus. These tumors may secrete ACTH, leading to the rapid development of hypercortisolism and ultimately Cushing's syndrome.^4^ Thymic malignancies are a rare group of diseases composing approximately 1% of all malignancies. Out of these malignancies, thymic neuroendocrine tumors comprise around 2%‐5% of the cases and are considered the least common.^4^ They inherently occur more in men, with a median age of diagnosis around the 5th decade of life.^5^ These are usually aggressive and malignant with the potential to metastasize; hence, treatment should be prompt to remove the thymus gland. The response for the treatment is variable depending on prognostic factors.^5^

## 2 CASE PRESENTATION

We report a 24‐year‐old Filipina woman who was recently diagnosed with hypertension and hypothyroidism. She presented to our hospital with generalized fatigue and weakness that had been going on for three months prior to presentation. She had increased abdominal girth along with acne and occasional difficulty while getting up from chairs or walking. On examination, she was found to be thinly built with a slightly round face containing numerous acne. A slight hump on her back was noted. She also had proximal bilateral muscle weakness, mainly in the lower limbs. Her blood pressure was found to be 163/67 mmHg. Laboratory tests revealed an 8 AM cortisol level to be high with high ACTH. A low‐dose dexamethasone suppression test failed to suppress cortisol (Table 1). At this point, pituitary magnetic resonance was performed, and it showed a pituitary microadenoma. Then, the high‐dose dexamethasone suppression test showed high cortisol levels (Table 1), which meant failure to suppress cortisol, suggesting the cause to be an ectopic source, rather than central.

**TABLE 1:** Hormonal Profile

Investigation	Result	Reference Range
Results before low Dose dexamethasone test		
8 AM cortisol level	2636 nmol/L	133‐537 nmol/L
ACTH level	114 pg/ml	7.2‐63.3 pg/ml
Results after low Dose dexamethasone test		
Cortisol	3572 nmol/L	133‐537 nmol/L
High dose dexamethasone test Cortisol	2959 nmol/L	133‐537 nmol/L

**FIGURE 1:**
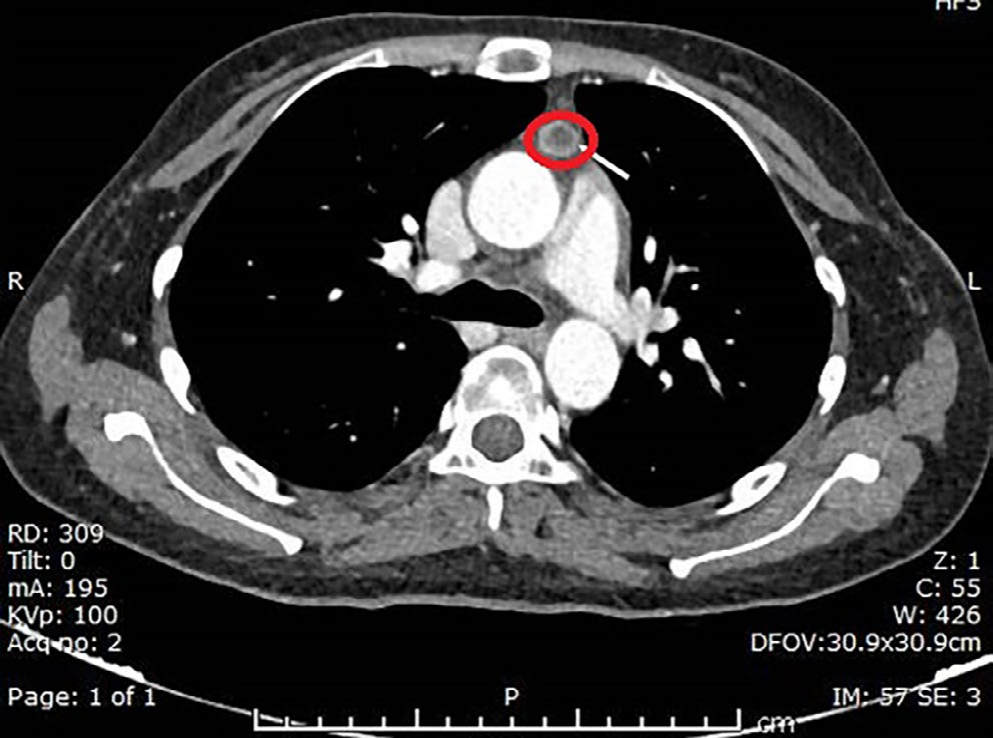
Axial contrast‐enhanced CT study at the level of midthoracic regions showing a small peripherally enhancing solid abnormality within the thymus circle

Chest, abdomen, and pelvic CT scans were taken; they showed findings of a small thymic neuroendocrine tumor (Figure 1). (Axial contrast‐enhanced CT study at the level of midthoracic regions shows a small peripherally enhancing solid abnormality within the thymus (circle)) associated with bilateral adrenal hypertrophy.

**FIGURE 2:**
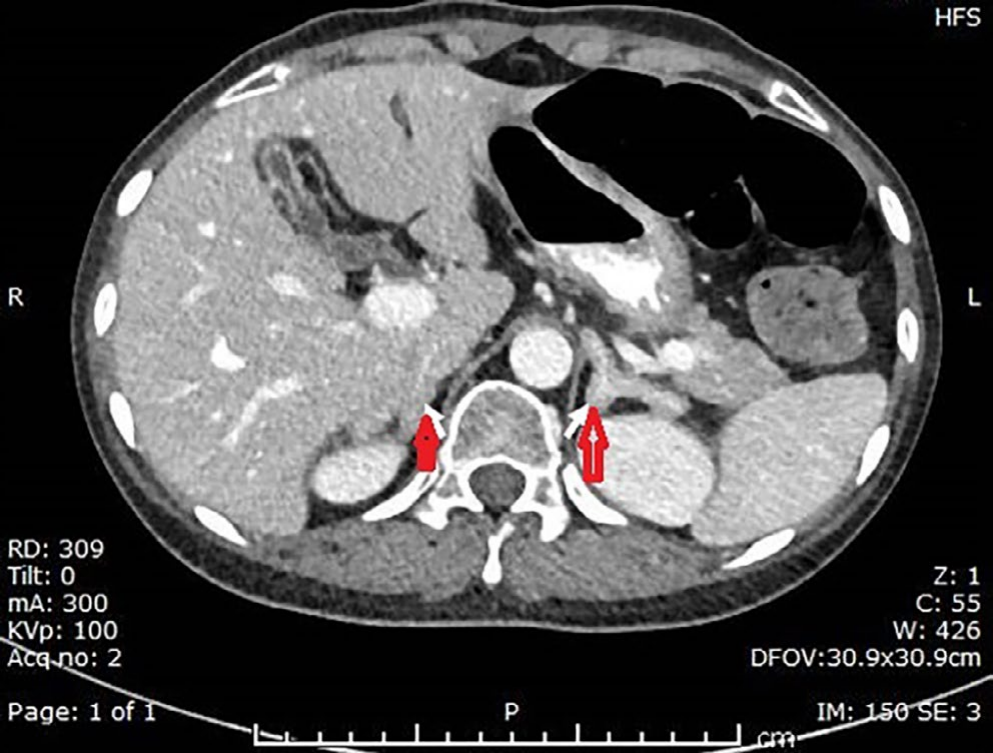
Axial contrast‐enhanced CT study at the level of the upper abdomen showing a smooth enlargement of both adrenal glands in keeping with hypertrophy (arrows)

Figure 2. (Axial contrast‐enhanced CT study at the level of the upper abdomen shows a smooth enlargement of both adrenal glands in keeping with hypertrophy (arrows)). This further confirmed the suspected diagnosis of Cushing's syndrome secondary to a thymic neuroendocrine tumor. She underwent surgical excision of the tumor by right video‐assisted thoracoscopic surgery (VATS) with en bloc excision of the mediastinal mass and total removal of anterior mediastinal fat.

**FIGURE 3:**
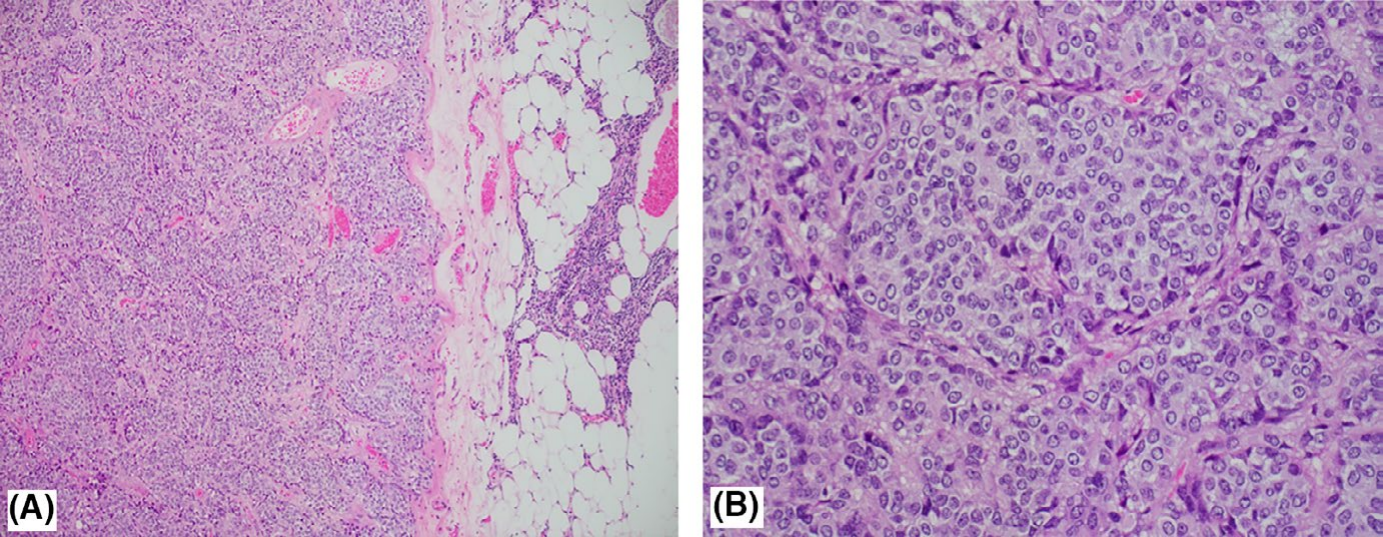
A, Light microscopic view showing the carcinoid tumor with well‐circumscribed tumor sharply demarcated from the uninvolved thymic tissue (H&E ×100). B, Light microscopic view showing tumor cells with round to oval nuclei and eosinophilic cytoplasm arranged in nests. The stroma is fine and vascularized (H&E ×400)

**FIGURE 4:**
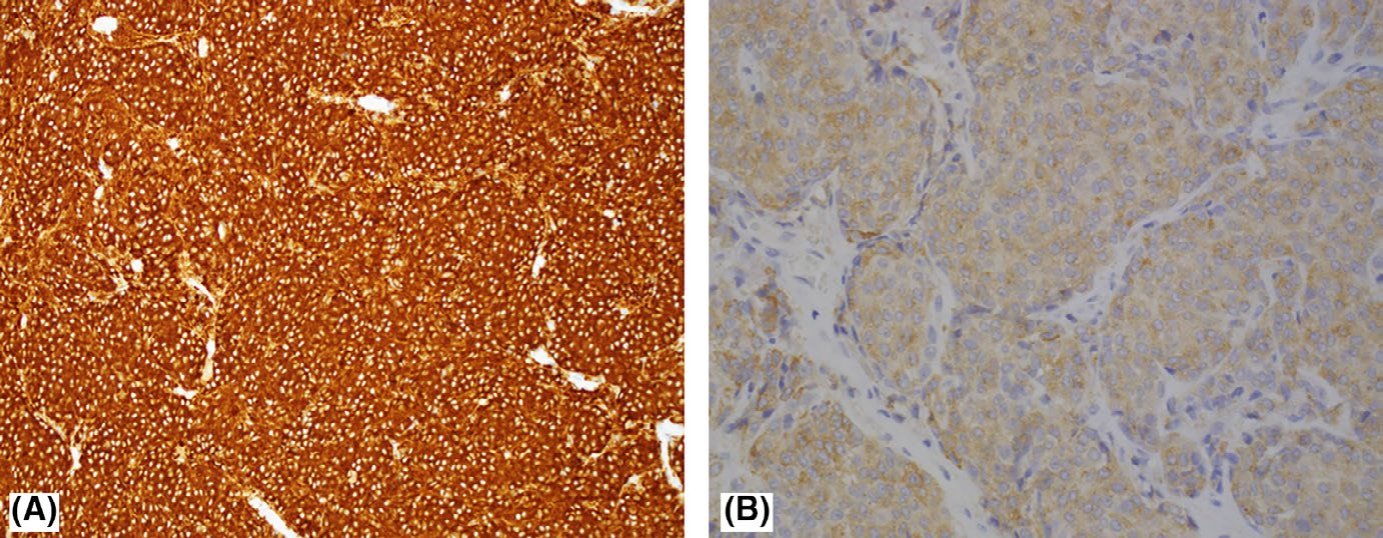
A, Immunohistochemical stain for synaptophysin highlighting the neuroendocrine cells (immunohistochemistry ×100). B, Immunohistochemical stain for ACTH shows focal faint staining of the neuroendocrine cells (immunohistochemistry ×200)

Pathological examination of the resected thymus revealed a well‐circumscribed tumor sharply demarcated from the surrounding atrophic thymic tissue (Figure 3 A). The tumor cells are arranged in a nested pattern. The cells are characterized as having round to oval nuclei, salt‐and‐pepper chromatin, and eosinophilic cytoplasm. The interstitium between the tumor cells is fine with small capillaries (Figure 3B). By immunohistochemistry, the tumor cells are positive with CKAE1/AE3 (paranuclear dot‐like pattern), synaptophysin, and ACTH (Figure 4A and 4B), and negative with chromogranin, TTF‐1, and PAX8. The Ki‐67 proliferative index is about 1%. The features were consistent with typical carcinoid tumor (ACTH expressing) confined to the thymus. Following the surgery, her ACTH dropped to 6.1 pg/m and cortisol to 399 nmol/L, as shown in Table 2, along with improvement in her symptoms and blood pressure and titration down on her antihypertensives with no postoperative complications.

**TABLE 2:** Hormonal Profile following VATS

Investigation	Result	Reference Range
8 AM Cortisol	399 nmol/L	133‐537 nmol/L
ACTH Levels	6.1 pg/m	7.2‐63.3 pg/ml

## 3 DISCUSSION

We present a rare case of Cushing's syndrome we encountered. The initial presentation was suggestive of endocrinopathy, which was confirmed by biochemical tests revealing hypercortisolism secondary to an ectopic source. The point of confusion was when the pituitary MRI showed a microadenoma, which led to the thinking that it might be the source; however, the high‐dose dexamethasone test was more pointing toward an ectopic source, rather than a central. One scan revealed a tiny tumor confined to the thymus. After resecting the tumor, the symptoms and laboratory findings related to Cushing's syndrome improved with no postoperative complications. On sequential follow‐ups, the patient had resolution of her symptoms and normalization of her blood pressure, which led to discontinuation of the antihypertensive medications. Also, her hormonal profile showed normalization of the cortisol levels.

There is little evidence in the literature describing Cushing's syndrome with a neuroendocrine thymic tumor.^6^ Thymic NETs are usually rare and account for a small percentage of malignancies, occurring more in male individuals at a ratio of 3:1.^7^ Functional thymic tumors tend to have a more complicated course and rapid onset of the development of clinical symptoms when associated with endocrinopathy. Suspicion of such tumors should be raised and sought when diagnosing an ectopic Cushing's syndrome. They are detected by imaging studies, such as CT scan of the chest, FDG PET, or Dotatate PET CT, which are also helpful in identifying any synchronous metastasis.^8^ Surgical excision remains the mainstay of treatment, and prognosis depends on the presence of metastasis and histopath logic parameters such as mitotic activity.^8^

## 4 CONCLUSION

While investigating the causes of Cushing's syndrome, ectopic sources should always be considered. A high clinical suspicion is needed. Thymic neuroendocrine tumors are considered malignant, with the diagnosis being dependent on biochemical, imaging, and pathological examinations for confirmation. The mainstay of treatment is surgical excision. The prognosis depends on several factors, the most important being early diagnosis and absence of distant metastasis. Long‐term monitoring is needed to oversee recurrence.

## CONFLICT OF INTEREST

None declared.

## AUTHOR CONTRIBUTIONS

Ahmad S matarneh: manuscript writing, data collection, and clinical care. Abdelrahman O Hamad: clinical care, study planning and design, and mentor. Mohammad Khair: clinical care. Elhadi ELouzi: clinical care. Mousa S Hussein: clinical care. Nabil Sherif Mahmood: clinical care and radiology contribution. Mohammad Yassin: mentor. Khaled Al‐Sawalmeh: pathology contribution. Issam Al‐Bozom: pathology contribution.

## ETHICAL APPROVAL

It was approved by Hamad Medical Corporation (MRC number MRC‐04‐20‐451).

## Data Availability

The authors confirm that the data supporting the findings of this study are available within the article and its supplementary materials.
